# Effects of electroacupuncture on urinary metabolome and microbiota in presenilin1/2 conditional double knockout mice

**DOI:** 10.3389/fmicb.2022.1047121

**Published:** 2023-01-24

**Authors:** Jie Gao, Nian Zhou, Mengna Lu, Qixue Wang, Chenyi Zhao, Jian Wang, Mingmei Zhou, Ying Xu

**Affiliations:** ^1^School of Rehabilitation Science, Shanghai University of Traditional Chinese Medicine, Shanghai, China; ^2^Department of Rehabilitation Medicine, Affiliated Hospital of Nantong University, Nantong, China; ^3^Institute for Interdisciplinary Medicine Sciences, Shanghai University of Traditional Chinese Medicine, Shanghai, China; ^4^Tongji University Cancer Center, Shanghai Tenth People’s Hospital, School of Medicine, Tongji University, Shanghai, China; ^5^School of Pharmacy, Shanghai University of Traditional Chinese Medicine, Shanghai, China; ^6^Department of Physiology, School of Basic Medicine, Shanghai University of Traditional Chinese Medicine, Shanghai, China

**Keywords:** electroacupuncture, PS cDKO mice, Alzheimer’s disease, metabolomics, gut microbiota

## Abstract

**Aim:**

The treatment of Alzheimer’s disease (AD) is still a worldwide problem due to the unclear pathogenesis and lack of effective therapeutic targets. In recent years, metabolomic and gut microbiome changes in patients with AD have received increasing attention, and the microbiome–gut–brain (MGB) axis has been proposed as a new hypothesis for its etiology. Considering that electroacupuncture (EA) efficiently moderates cognitive deficits in AD and its mechanisms remain poorly understood, especially regarding its effects on the gut microbiota, we performed urinary metabolomic and microbial community profiling on EA-treated AD model mice, presenilin 1/2 conditional double knockout (PS cDKO) mice, to observe the effect of EA treatment on the gut microbiota in AD and find the connection between affected gut microbiota and metabolites.

**Materials and methods:**

After 30 days of EA treatment, the recognition memory ability of PS cDKO mice was evaluated by the Y maze and the novel object recognition task. Urinary metabolomic profiling was conducted with the untargeted GC-MS method, and 16S rRNA sequence analysis was applied to analyze the microbial community. In addition, the association between differential urinary metabolites and gut microbiota was clarified by Spearman’s correlation coefficient analysis.

**Key findings:**

In addition to reversed cognitive deficits, the urinary metabolome and gut microbiota of PS cDKO mice were altered as a result of EA treatment. Notably, the increased level of isovalerylglycine and the decreased levels of glycine and threonic acid in the urine of PS cDKO mice were reversed by EA treatment, which is involved in glyoxylate and dicarboxylate metabolism, as well as glycine, serine, and threonine metabolism. In addition to significantly enhancing the diversity and richness of the microbial community, EA treatment significantly increased the abundance of the genus *Mucispirillum*, while displaying no remarkable effect on the other major altered gut microbiota in PS cDKO mice, *norank_f_Muribaculaceae*, *Lactobacillus*, and *Lachnospiraceae_NK4A136 group*. There was a significant correlation between differential urinary metabolites and differential gut microbiota.

**Significance:**

Electroacupuncture alleviates cognitive deficits in AD by modulating gut microbiota and metabolites. *Mucispirillum* might play an important role in the underlying mechanism of EA treatment. Our study provides a reference for future treatment of AD from the MGB axis.

## 1. Introduction

Alzheimer’s disease (AD) is a neurodegenerative disease, mainly manifested as memory impairment, apraxia, agnosia, impaired spatial ability, computing power, and personality and behavior changes. AD has become the third leading cause of disability and death in the elderly, only after cardiovascular and cerebrovascular diseases and malignancies (Du et al., 2018). There are various hypotheses about its etiology: abnormal deposition of amyloid beta (Aβ) in the extracellular space of neurons, the formation of tau protein tangential fibers in neurons, inflammation, cholinergic neuron damage, oxidative stress, etc. But it is hard to explain the disease entirely with one hypothesis. Over the past decade, it has been widely believed that microbiome changes are closely related to neurodegenerative diseases, of which AD is one of the most representative diseases. The gut microbiota of different AD transgenic mice has been reported to vary with age, implying an association with disease progression (Zhang L. et al., 2017; Wang et al., 2019). Similarly, the composition and diversity of microbiota in fecal samples from patients with AD also changed compared with healthy subjects (Zhuang et al., 2018). The microbiome–gut–brain (MGB) axis, which has been used to describe direct or indirect relations among the brain, gut, and gut microbiota, is primarily bidirectional crosstalk through three distinct but parallel communication pathways “neuro-immune-endocrine” (Du et al., 2018; Sun M. et al., 2020).

Several recent studies have enriched the evidence of changes in the MGB axis in AD pathogenesis, which may explain various characteristics of AD processes along with changes in the gut microbiota. In APPS_WE_/PS1_Δ*E*9_ transgenic mice, antibiotic-induced disturbance of gut microbial diversity shows an effect on Aβ plaque deposition and neuro-inflammation (Minter et al., 2016). In addition, *Lactobacillus plantarum* contributed to reinforce the beneficial effects of memantine treatment in APP/PS1 mice by remodeling the gut microbial composition, inhibiting the synthesis of trimethylamine-N-oxide, a gut microbial metabolite, and reducing cluster protein levels. It is also observed in this research that improved cognitive deterioration, reduced Aβ levels in the hippocampus, and protected neuronal integrity and plasticity of the mice (Wang et al., 2020). Furthermore, oral probiotics to modify the gut microbiota has been proven to be beneficial in reducing oxidative stress (Bonfili et al., 2018) and restoring glucose homeostasis in 3xTg-AD mice (Bonfili et al., 2019), and abnormal glucose metabolism is also one of the most important clinical and biochemical characteristics leading to AD (Adlimoghaddam et al., 2019).

Metabolites derived from the gut microbiota play important roles in the MGB axis. For example, gut microbiota-derived short-chain fatty acids, *for example*, valeric acid, butyric acid, and propionic acid can interfere with Aβ aggregation (Ho et al., 2018). Similarly, metabolites released from abundant bacteria in a healthy gut such as 3-hydroxybenzoic acid and 3-(3′-hydroxyphenyl) propionic acid (Wang et al., 2015) support cognitive function, whereas metabolites released by pro-inflammatory bacteria in AD aggravated the inflammation of the central nervous system (Bostanciklioglu, 2019). Moreover, a growing number of studies have clearly shown various changes in the metabolism of AD, including cerebral glucose metabolism (Cisternas and Inestrosa, 2017; Adlimoghaddam et al., 2019), lipid metabolism (Han et al., 2011; Liao et al., 2017), and the metabolism of several amino acids in the dopamine-norepinephrine pathway (Kaddurah-Daouk et al., 2011). Metabolomic approaches can be available for qualitative and quantitative analyses of metabolic profiles. Compared with other biological fluids, the urine sample can be obtained non-invasively, contains abundant metabolites, and reflects the imbalance of all biochemical pathways in the body (Khamis et al., 2017). Urine metabolomics can detect subtle metabolic differences in specific diseases or therapeutic interventions.

Electroacupuncture (EA), a traditional treatment originating from China, is accepted worldwide now. The efficacy of EA for cognitive deficits in AD has been widely reported in clinical and animal studies (Peng et al., 2017; Cai et al., 2019). Some mechanisms have been documented, such as inhibition of neuroinflammation (Cai et al., 2019), improvement of N-acetylaspartate, glutamate and glucose metabolism (Liu et al., 2017; Lin et al., 2018), reduction of Aβ deposits (Li X. et al., 2014; Tang et al., 2019), upregulation of the expression of BDNF and promotion of neurogenesis (Li X. et al., 2014; Lin et al., 2016), activation of PPAR-γ (Zhang M. et al., 2017), and attenuation of NOX2-related oxidative stress (Wu et al., 2017). However, its biological basis is still unclear, and the link between its effect on AD and gut microbiota has rarely been reported. As mentioned earlier, the gut microbiota plays a very important role in the progression of AD. Therefore, it is very necessary to clarify the potential role of EA in AD, especially from the gut microbiota and metabolomics.

Presenilin 1/2 conditional double knockout (PS cDKO) mice have been widely accepted as mice with a typical phenotype of AD (Saura et al., 2004; Lee and Aoki, 2012; Zhao et al., 2019). They exhibit age-dependent AD-like symptoms and pathology, such as cognitive deficits and synaptic plasticity impairments from the early stage, obvious neuroinflammation at a mature age, hyperphosphorylated tau, and cortical and hippocampal atrophy in the late stage (Saura et al., 2004; Chen et al., 2008; Zhao et al., 2019). Moreover, our previous studies have demonstrated that they displayed metabolic and microbiotic changes, which were associated with the progression of the disease (Gao et al., 2021b). Considering all these, in this work, we aim to investigate the influence of EA on the metabolome and gut microbiota in PS cDKO mice and further find out the potential mechanism by which EA acts on cognition under gut microbial regulation.

## 2. Materials and methods

### 2.1. Animals

The generation and genotyping of PS cDKO mice have been described previously (Saura et al., 2004). Mice with the transgene Cre, fPS1/fPS1, and PS2-/- were performed as PS cDKO mice, whereas their littermates, without transgene Cre, fPS1/+, and PS2+/+, or PS2±, assigned to the wild-type (WT) group. All mice were housed in a specific pathogen-free environment since born with food and water freely available. The room with 12-h light/dark cycles was controlled at 23 ± 2°C. All animal protocols in this study were approved by Animal Experimentation of Shanghai University of Traditional Chinese Medicine (PZSHUTCM191025005) and carried out in accordance with relevant guidelines and regulations. All methods are also in accordance with the ARRIVE guidelines.

### 2.2. Electroacupuncture treatment and sample collection

Five months of PS cDKO mice were randomly assigned into the cDKO group and the cDKO with EA treatment (cDKO + EA) group (*n* = 6). Mice between groups were sex-matched, with half males and half females. Mice in each group were housed separately to avert the cage effects from microbiome transfer. For the cDKO + EA group, disposable acupuncture needles (0.17 mm × 7 mm, Changchun AIK Medical Device Co., Ltd., Changchun, China) were inserted perpendicularly into the muscle layer at Shenmen (HT7) and Taixi (KI3) on the same side limbs. The Shenmen acupoint is located on the ulnar end of the carpal transverse grain in the forepaw, while Taixi is on the midpoint between the Achilles tendon and the medial malleolus. The bilateral acupoints were used alternately in the treatment. The pulse generator (G6805, Shanghai Medical Instrument High Technology Co., Ltd., Shanghai, China) was connected to deliver electrical current to the needles (continuous wave: 2 Hz, 1 mA, lasted 15 min). EA stimulation was administered every day starting at 8 a.m. and lasted for 30 days. The cDKO group mice and WT group received the same type of fixation for equal time. After that, fecal samples were collected. For the collection of urine, each mouse was separately kept in metabolic cages for 1 day during the fasting state. The whole procedure was also conducted in a specific pathogen-free environment. The fecal and urine samples were snap-frozen in liquid nitrogen, and then stored at −80°C before further analysis.

### 2.3. Behavioral tests

To observe whether EA has an influence on cognitive deficits in PS cDKO mice, a Y maze and a Novel object recognition task (Gao et al., 2021a) were conducted successively at a 3-day interval. Mice were placed in a sound-proofed behavior room in advance to adapt to the circumstance. The operators were blind to the condition of each mouse for behavioral tests.

#### 2.3.1. Y maze

The Y maze, used to assess spatial recognition memory ability, was conducted as previously described (Gao et al., 2021a). First, one arm, named the novel arm, was blocked. Each mouse was placed in the start arm, facing the central joining region, and allowed to freely explore the opened two arms for 8 min. One hour later, the novel arm was opened and the mouse was replaced in the start arm to freely explore the three arms. The percentage of time mice spent and the number of entries in the novel arm were calculated.

#### 2.3.2. Novel object recognition task

The novel object recognition task including three sessions is also used to evaluate recognition memory ability. During the first training sessions, an open-field chamber was set up with two objects of the same size, shape, color, and material. Each mouse was placed in it to explore for 5 min. At 1 h and 24 h after the training sessions, the mouse was placed in the chamber again, but one of the objects was replaced with a different object in size, color, and shape. The time that each mouse spent exploring each object was recorded. The ratio of time that mice spent exploring either of the same objects (during the training session) or the novel object (the next two sessions) over the total time that mice spent exploring both objects was calculated as the preference index.

### 2.4. Urinary metabolomic signatures

Urine metabolites were performed following an untargeted gas chromatography-time-of-flight mass spectrometer (GC-MS) metabolomics method, as described previously (Ma et al., 2018). In brief, urinary samples were first thawed at room temperature, shaken well, and then centrifuged at 12,000 rpm for 10 min. One hundred microliter of supernatant was taken and mixed with 70 IU urease for 15 min for urea degeneration, and then methanol and myristic acid were added. The supernatant was centrifuged and dried using a nitrogen stream. The prepared methoxide was then combined with the carbonyl group by adding pyridine-dissolved methoxyamine. After that, NO-Bis (trimethylsilyl) trifluoroacetamide acted as a derivatizing reagent to pretreatment. The Agilent 6890/5975B GC/MSD system was used to perform the sample analysis. Each 1 μL analyte was injected into a capillary column (Agilent J&W DB-5ms Ultra Inert 30 m × 250 μm, i.d., 0.25 μm film thickness) with high purity helium as carrier gas at a constant flow rate of 1.0 ml/min. The solvent delay time was set to 5 min. Temperature programing for GC was set at 70°C for 2 min and followed by a 2.5°C/min oven temperature ramp to 160°C, then raised to 240°C at a rate of 5°C/min, and maintained at that temperature for 16 min. The temperatures of the injector, the EI iron source, and the interface were set to 280, 230, and 260°C, respectively. The measurements were collected using electron impact ionization (70 eV) in full scan mode (m/z 50–600).

### 2.5. Microbial community profiling

Total microbial DNA was extracted from fecal contents using the E.Z.N.A.^®^ soil DNA kit (Omega Bio-Tek, Norcross, GA, USA), according to standard protocols. Gel electrophoresis (0.8% agarose gel) was used for DNA extraction, and then an ultraviolet spectrophotometer (Thermo Fisher Scientific, Wilmington, USA) was used to evaluate DNA concentration and purity. To amplify the hypervariable regions (V3–V4) of the bacterial 16S ribosomal RNA gene, a set of primers (338F: 5′-ACTCCTACGGGAGGCAGCAG-3′ and 806R: 5′-GGACTACHVGGGTWTCTAAT-3′) was used. Polymerase chain reaction (PCR) amplification products were identified by 2% agarose gel electrophoresis and then purified by the AxyPrep DNA Gel Extraction kit (AXYGEN Biosciences, Union City, CA, USA). The purified amplicons were combined at an equimolar ratio, which was quantified using a microplate reader (BioTek, FLx800, USA). Finally, the paired terminal sequencing was performed on the Illumina MiSeq platform under the standard instructions (Majorbio Biopharm Technology Co., Ltd., Shanghai, PRC).

### 2.6. Data analysis

First, raw data analyzed by GC-MS were converted into NetCDF format by Agilent MSD workstation. Then, XCMS toolkit scripts and R 2.13.2 (Lucent Technology, Reston, VA, USA) packages are used for preprocessing, and subsequently, Simca 14 software (Umetrics, Umea, Sweden) was used for further processing. Raw FASTQ files were demultiplexed and qualified by fastp version 0.20.0 and merged by FLASH version 1.2.7 as we described previously (Gao et al., 2021b). In brief, the 300 bp reads were truncated at any site getting an average quality score of lower than 20 over a 50 bp sliding window. Only overlapping sequences of >10 bp were assembled and the maximum mismatch ratio of the overlap region is 0.2. Besides, distinguish samples based on barcode and primers and only accept two nucleotide mismatch in primer matching. Operational taxonomic units (OTUs) picked at 97% similarity cutoff were clustered using UPARSE version 7.1. The taxonomy of each OTU representative sequence was analyzed by RDP Classifier version 2.2 with a confidence threshold of 0.7. Differences in α-diversity were computed by the Shannon and Chao indices. β-diversity was performed to estimate the difference or similarity of community structure between groups, visualized in principal coordinate analysis (PCoA) plots. The statistical significance was assessed by partial least squares discriminant analysis (PLS-DA). Different bacterial taxa among groups were estimated by the linear discriminant analysis (LDA) effect size (LEfSe).

Statistical analyses were carried out with a one-way ANOVA, the two-tailed Student’s *t*-test, or Pearson’s multivariate linear regression analysis by SPSS 25.0. The correlation coefficient of Spearman’s between perturbed urinary metabolome and gut taxa was indicated as a heatmap. All numerical data are shown as means ± standard deviation (SD). *P* < 0.05 was considered statistically significant. The metabolic associations of each well-correlated member of the gut microbe (|*r*| > 0.4) were considered as a cross-correlation diagram.

## 3. Results

### 3.1. Electroacupuncture ameliorates cognitive deficits in PS cDKO mice

**FIGURE 1:**
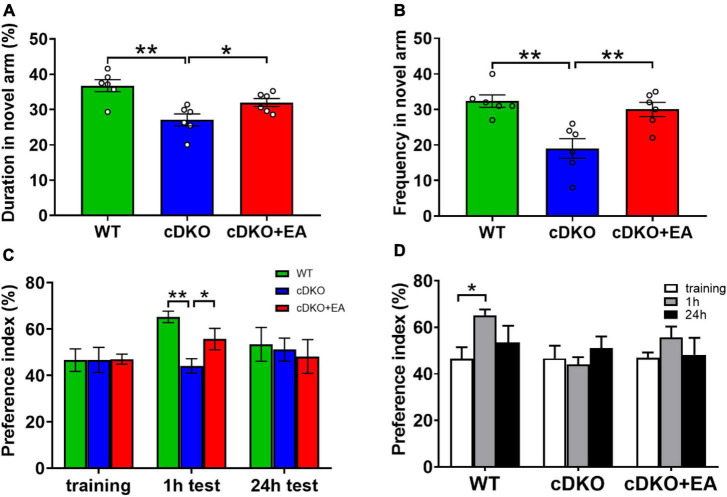
Electroacupuncture ameliorates cognitive deficits in PS cDKO mice. The duration **(A)** and frequency **(B)** of entries in the novel arm of the Y maze. The symbol “°” means an individual. **(C,D)** The preference index in the novel object recognition task. One-way ANOVA, *P < 0.05, ^**^P < 0.01, *N* = 6.

PS cDKO mice is a generally accepted AD model, characterized by cognitive deficits, which is accordant with our study. It is well known that mice prefer to explore new things. In the Y maze, compared with WT controls, PS cDKO mice showed significantly reduced duration and frequency in the novel arm, which was blocked at first, indicating reduced spatial recognition memory. However, EA treatment improved the duration and frequency observed in the novel arm (Figures 1A, B). Interestingly, similar cases happened in the novel object recognition task. During the training session, mice did not exhibit different preferences for the two similar objects in groups (Figure 1C). At 1 h after the training session (for testing short-time memory), WT mice displayed an obvious preference for the novel object, while PS cDKO mice did not spend much time exploring the novel object (Figures 1C, D). Though there was such preference neither in WT nor in PS cDKO mice in subsequent 24 h tests (for long-time memory), those suggested that PS cDKO mice had impaired short-time novel object recognition memory. However, the time spent on exploring the novel object in 1 h tests was reversed by EA treatment.

All those indicated that EA ameliorated short-term memory deficits.

### 3.2. Electroacupuncture alters urinary metabolome in PS cDKO mice

**FIGURE 2:**
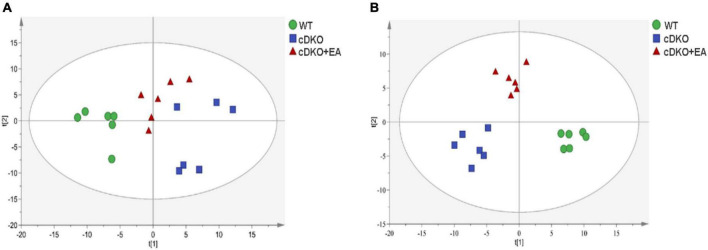
Scores plots of multivariate statistical analysis on urinary metabolites. PCA scores plot **(A)** and PLS-DA scores plot **(B)** of the WT, cDKO, and cDKO + EA groups. *N* = 6.

By establishing principal component analysis (PCA) and PLS-DA patterns, we observed overall clustering and trends among groups. As shown in Figure 2A, significant separation was observed among WT, cDKO, and cDKO + EA groups in the PCA score plot (*R*^2^X = 0.821, *Q2* = 0.508). Moreover, in the PLS-DA score plot (Figure 2B), the cDKO group was separated from the WT and cDKO + EA groups (*R*^2^X = 0.864, *R*^2^Y = 0.982, *Q*^2^ = 0.959). The result indicated that the model was constructed successfully, and EA treatment appeared to ameliorate urine metabolic alternation induced by cDKO. In the OPLS-DA plot (Supplementary Figures 1A, B), the WT and DKO groups displayed significant deviation (*R*^2^X = 0.689, *R*^2^Y = 0.992, *Q^2^* = 0.918). Supplementary Figure 1C also showed that the cDKO + EA group had distinctive metabolic profiles compared with the cDKO group (*R*^2^X = 0.639, *R*^2^Y = 0.956, *Q^2^* = 0.798).

**FIGURE 3:**
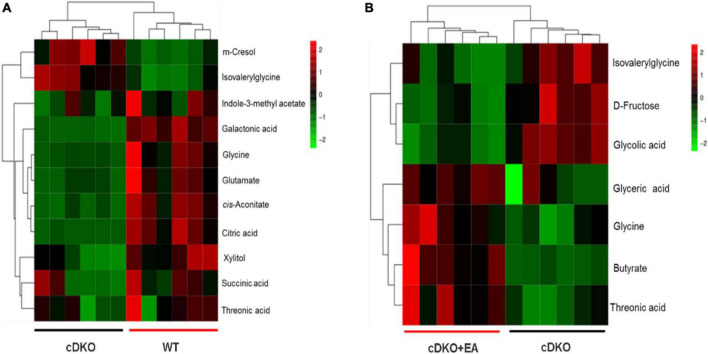
Heat map of the differential metabolites in the cDKO group and the WT group **(A)**, and the cDKO group and the cDKO + EA group **(B)**, *N* = 6.

Screening and identification of differential metabolites using S-plot and VIP (variable importance in projection) in OPLS-DA (VIP > 1), which were further verified by a pairwise *t*-test (*P* < 0.05) (Xue et al., 2021). As shown in Figure 3A, a total of 11 differential metabolites were identified in the WT group compared with the cDKO group in which m-cresol and isovalerylglycine decreased in the WT group, and the other nine differential metabolites increased in the WT group. At the same time, we also observed the abundance of those 11 differential metabolites in the cDKO + EA group (Supplementary Figure 1E). Compared with the cDKO group, the cDKO + EA group showed an obvious decrease in isovalerylglycine and increased glycine and threonic acid with the other eight metabolites changed insignificantly (Supplementary Figure 1E and Figure 3B). Moreover, there was a total of seven different metabolites were identified between the cDKO and cDKO + EA groups of which three metabolites were reduced in the cDKO + EA group (Figure 3B). The details of these metabolites are presented in Supplementary Tables 1, 2.

### 3.3. Electroacupuncture affects the metabolic pathway and network analysis

**FIGURE 4:**
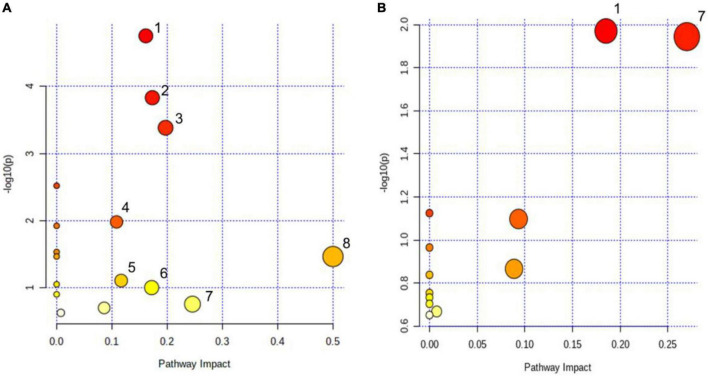
Pathway analysis of urinary metabolites using metaboanalyst (impact factor ≥0.1). **(A)** Disturbed metabolic pathways in PS cDKO mice. **(B)** Influenced metabolic pathways of PS cDKO mice after electroacupuncture. (1) Glyoxylate and dicarboxylate metabolism, (2) citrate cycle (TCA cycle), (3) alanine, aspartate, and glutamate metabolism, (4) glutathione metabolism, (5) arginine biosynthesis, (6) pentose and glucuronate interconversions, (7) glycine, serine, and threonine metabolism, and (8) d-glutamine and d-glutamate metabolism. *N* = 6.

KEGG and HMDB databases were employed to correlate urine differential metabolites with potentially related pathways, and MetaboAnalyst 3.0 was used to further determine their impact values. It was found that the eight most relevant metabolic pathways were disturbed (impact factor ≥0.1), compared the cDKO group with the WT group. They were glyoxylate and dicarboxylate metabolism; citrate cycle (TCA cycle); alanine, aspartate, and glutamate metabolism; glutathione metabolism; arginine biosynthesis; pentose and glucuronate interconversions; glycine, serine, and threonine metabolism; d-glutamine and d-glutamate metabolism (Figure 4A). Moreover, two main affected metabolic pathways were observed in the cDKO and cDKO + EA groups. They are glyoxylate and dicarboxylate metabolism and glycine, serine, and threonine metabolism (Figure 4B). In addition, there were two identical metabolic pathways involved in three groups, suggesting that the two pathways may have an important role in the pathological process of AD and the course of EA treatment.

### 3.4. Electroacupuncture changes the gut microbiome in PS cDKO mice

**FIGURE 5:**
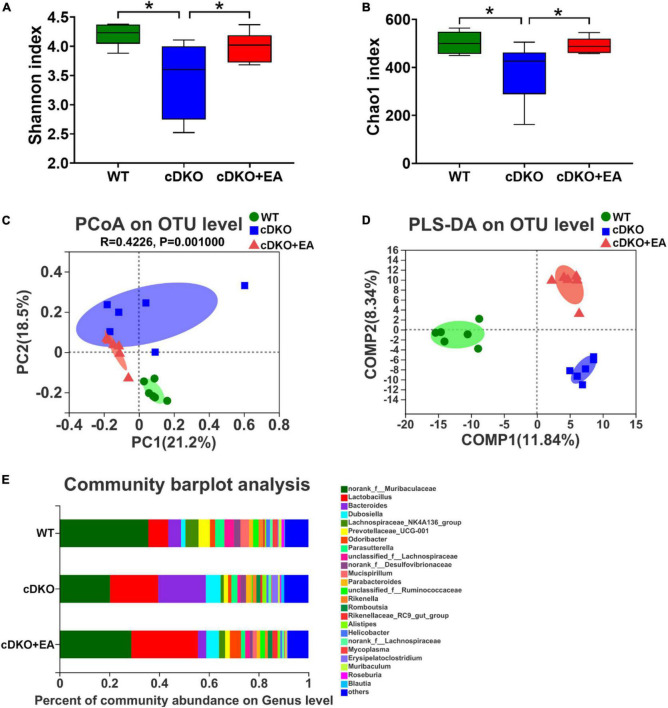
Influence of electroacupuncture on gut taxa in PS cDKO mice. The α-diversity indexes of Shannon **(A)** and Chao1 **(B)** of gut microbiota. *P < 0.05. PCoA **(C)** and PLS-DA **(D)** of the gut microbiome composition of WT, cDKO, and cDKO + EA groups on the OUT level. **(E)** Relative abundance of the gut microbiome in the three groups, colored at and genus level, *N* = 6.

Our previous study has observed that the gut microbiome has been changed in PS cDKO mice (Gao et al., 2021b), but the effect of EA on it is still unclear. The α-diversity indexes including Shannon and Chao1 were conducted to reflect the community diversity and richness in three groups. The Shannon index shows PS cDKO mice have reduced diversity within the microbial community; however, it was reversed after PS cDKO mice were treated with EA (Figure 5A). The same trend happened in the Chao1 index (Figure 5B), showing EA improving community richness of PS cDKO mice. Furthermore, PcoA and PLS-DA demonstrated the obviously different community structure of the gut microbiome between PS cDKO mice and WT mice, and the community structure changed after PS cDKO mice received EA (Figures 5C, D). Especially, in PcoA, the community structure in the cDKO + EA group was more close to the WT group. Furthermore, we studied the community abundance of the gut microbiome in three groups, which could show the microbiotic community compositions intuitively. The genus microbiota, *Lactobacillus*, *Bacteroides*, and *Dubosiella* were the three most prominent microbiotic communities in three groups, followed by *Lachnospiraceae_NK4A136_group* and *Prevotellaceae_UGG-001*, and the abundance of those three microbiomes was increased in PS cDKO mice (Figure 5E). After EA, the total abundance of those was decreased, while the abundance of *Lactobacillus* and *Bacteroides* showed confused fluctuation. In addition, PS cDKO mice displayed declined *Lachnospiraceae_NK4A136_group* and *Prevotellaceae_UGG-001*. Their relative abundance was reversed but not very obviously in PS cDKO mice treated by EA. These results suggested that EA upgraded diversity and richness within the microbial community and changed the community structure in PS cDKO mice, with the abundance of part changed main microbiota moderated.

### 3.5. Electroacupuncture moderates the structure of gut taxa in PS cDKO mice

**FIGURE 6:**
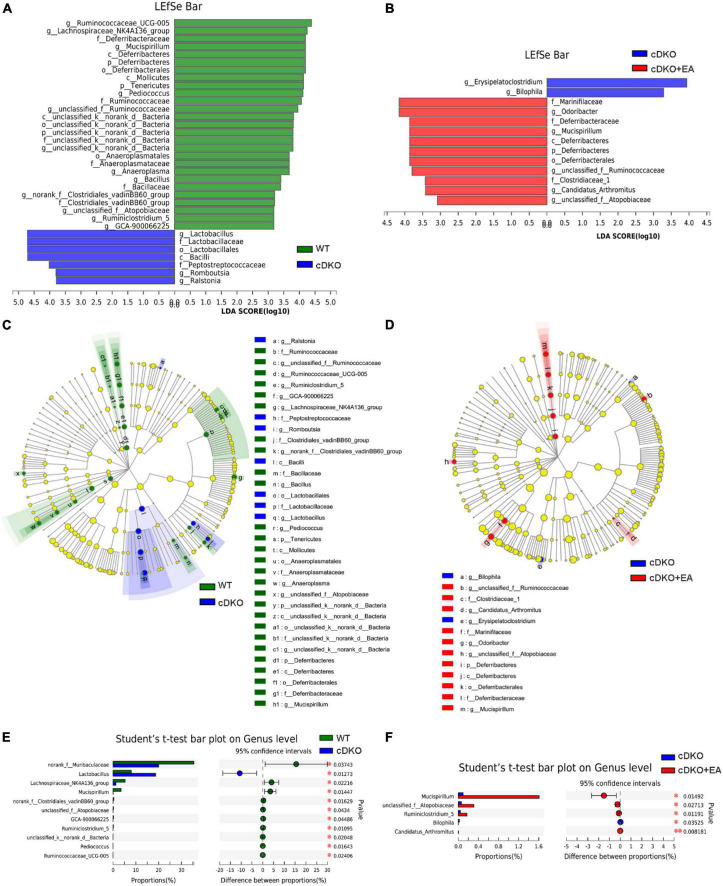
The taxa of gut microbiota affected by electroacupuncture. LEfSe analysis from the phylum to genus level in the WT and cDKO groups **(A)**, the cDKO and cDKO + EA groups **(B)**. Taxa enriched in three groups are indicated by LDA scores (green for the WT group, blue for the cDKO group, and red for the cDKO + EA group). The LDA score threshold is ≥3. **(C,D)** The cladogram of enriched taxa from the phylum to genus level. Differential abundance analysis of taxa on the genus level in the WT group and the cDKO group **(E)**, the cDKO group, and the cDKO + EA group **(F)**. Student’s *t*-test, *P < 0.05, ^**^P < 0.01, *N* = 6.

To clarify the exact change in the structure of gut taxa, we conducted LEfSe analysis to compare the gut microbiota in different groups at diverse taxonomic levels. LEfSe analysis revealed PS cDKO mice mainly showed a higher abundance of *g_Lactobacillus*, *f_Lactobacillaceae*, *o_Lactobacillales*, *c_Bacilli*, *f_Peptostreptococcaceae*, *g_Romboutsia*, and *g_Ralstonia* (LDA score of ≥3) from phylum to genus (Figure 6A); however, WT mice were largely characterized by higher enrichment of 27 other microbiota. Furthermore, compared with PS cDKO mice, PS cDKO mice treated with EA were featured by higher enrichment of *f_Marinifilaceae*, *g_Odoribacter*, *f_Deferribacteraceae*, *g_Mucispirillum*, *c_Deferribacteres*, *p_Deferribacteres*, *o_Deferribacterales*, *g_unclassified_f_Ruminococcaceae*, f_Clostridiaceae_1, *g_Candidatus_Arthromitus*, and *g_unclassified_f_Atopobiaceae* (LDA score of ≥3) (Figure 6B). The hierarchical relationships among the enriched taxa in each group were exhibited on the cladogram clearly (Figures 6C, D). *G_Lactobacillus*, *f_Lactobacillaceae*, and *o_Lactobacillales*, the three most enriched taxa in PS cDKO mice, are all the subsets of *c_Bacilli*, and *g_Lactobacillus* are the next hierarchies of *f_Lactobacillaceae* (Figure 6C). The relationship of main taxa in PS cDKO mice with EA treatment from *g_Mucispirillum to p_Deferribacteres* was also well demonstrated (Figure 6D). In addition, we analyzed the change in the gut microbiota between the groups at the genus level. As shown in Figure 5E, the cDKO group had 11 gut taxa changes when compared with the WT group. *Norank_f_Muribaculaceae*, *Lactobacillus*, *Lachnospiraceae_NK4A136_gruop*, and *Mucispirillum* were the primary different taxa, obviously declined except *Lactobacillus*. Moreover, the proportions of five taxa were significantly different in the cDKO + EA group. The proportions of *Mucispirillum*, *unclassified_f_Atopobiaceae*, and *Ruminiclostridium_5*, the dominant taxa, were significantly increased. Taking all these into consideration, we could conclude that EA on PS cDKO mice significantly affects gut taxa and *Mucispirillum* may be the cue microbiota in the changes.

### 3.6. Correlation analysis of metabolomic signatures and microbiotic community profilings

**FIGURE 7:**
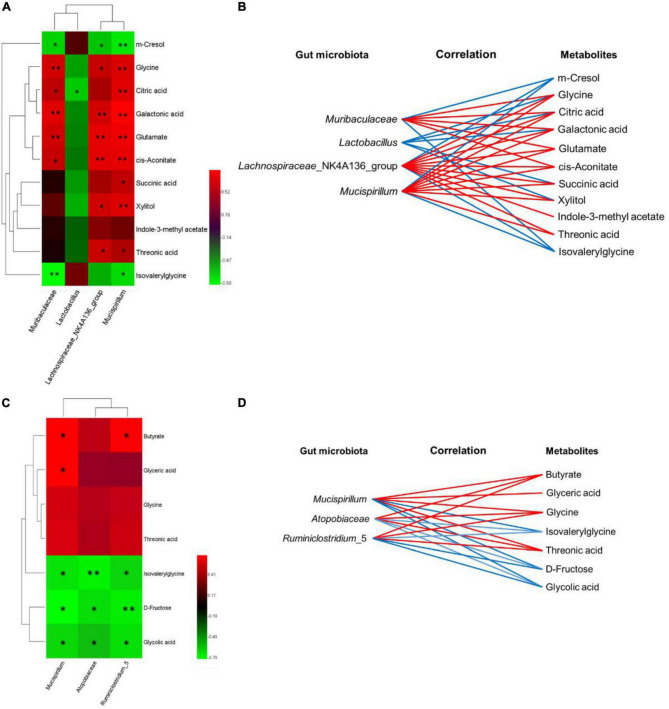
The relevance between the gut microbiota of genus level and the differential urinary metabolites. **(A–C)** Spearman’s correlation heat map: red indicates a positive correlation, while green indicates a negative correlation. The deeper color means a greater correlation (*P < 0.05, ^**^P < 0.01). **(B–D)** The gut microbiota of the genus level, predicted by metabolic variation (|r| > 0.4), is labeled with a similar value. Lines connecting with metabolites show the direction of the relevance to each genus of microbe with the red (positive) or blue (negative) lines, *N* = 6.

The covariant relationships between urinary differential metabolites and genus-level differential gut microbiological communities were multiple, which are represented by heatmaps (Figures 7A, C), and the metabolic connections of well-predicted bacteria were shown in Figures 6B, D (|*r*| > 0.4). In the WT and cDKO groups, *Muribaculaceae* was negatively correlated with m-cresol (*r* = −0.657, *P* < 0.05) and isovalerylglycine (*r* = −0.797, *P* < 0.01), and positively correlated with glycine (*r* = 0.713, *P* < 0.01), cis-aconitate (*r* = 0.671, *P* < 0.05), citric acid (*r* = 0.643, *P* < 0.05), galactonic acid (*r* = 0.762, *P* < 0.01), and glutamate (*r* = 0.713, *P* < 0.01). *Lactobacillus* has a negative correlation with glycine (*r* = −0.497), succinic acid (*r* = −0.469), xylitol (*r* = −0.538), citric acid (*r* = −0.629, *P* < 0.05), and galactonic acid (*r* = −0.413). *Lachnospiraceae_NK4A136_group* was negatively correlated with m-cresol (*r* = −0.608, *P* < 0.05) and isovalerylglycine (*r* = −0.538), and positively correlated with glycine (*r* = 0.671, *P* < 0.05), succinic acid (*r* = 0.559), threonic acid (*r* = 0.685, *P* < 0.05), glutamate (*r* = 0.776, *P* < 0.01), indole-3-methylacetate (*r* = 0.462), xylitol (*r* = 0.706, *P* < 0.05), cis-aconitate (*r* = 0.769, *P* < 0.01), citric acid (*r* = 0.573), and galactonic acid (*r* = 0.713, *P* < 0.01). *Mucispirillum* has a negative correlation with m-cresol (*r* = −0.720, *P* < 0.01) and isovalerylglycine (*r* = −0.685, *P* < 0.05) but has a positive correlation with glycine (*r* = 0.741, *P* < 0.01), succinic acid (*r* = 0.650, *P* < 0.05), threonic acid (*r* = 0.601, *P* < 0.05), glutamate (*r* = 0.804, *P* < 0.01), xylitol (*r* = 0.748, *P* < 0.01), cis-aconitate (*r* = 0.783, *P* < 0.01), citric acid (*r* = 0.762, *P* < 0.01), and galactonic acid (*r* = 0.853, *P* < 0.01). Moreover, in the cDKO and cDKO + EA groups, *Muribaculaceae* was positively correlated with butyrate (*r* = 0.706, *P* < 0.05), glycine (*r* = 0.531), threonic acid (*r* = 0.559), and glyceric acid (*r* = 0.706, *P* < 0.05), and negatively correlated with isovalerylglycine (*r* = −0.706, *P* < 0.05), D-fructose (*r* = −0.790, *P* < 0.01), and glycolic acid (*r* = −0.678, *P* < 0.05). *Atopobiaceae* has a positive correlation with butyrate (*r* = 0.458), glycine (*r* = 0.430), threonic acid (*r* = 0.401), and has a negative correlation with isovalerylglycine (*r* = −0.754, *P* < 0.01), D-fructose (*r* = −0.697, *P* < 0.05), and glycolic acid (*r* = −0.599, *P* < 0.05). *Ruminiclostridium_5* was positively correlated with butyrate (*r* = 0.685, *P* < 0.05), glycine (*r* = 0.476), and threonic acid (*r* = 0.503), and negatively correlated with isovalerylglycine (*r* = −0.671, *P* < 0.05), D-fructose (*r* = −0.741, *P* < 0.01), and glycolic acid (*r* = −0.699, *P* < 0.05). Together, these results indicated that differential gut microbiota was correlated with differential urinary metabolites.

## 4. Discussion

The disorder of the gut microbiome is an important factor to urge the pathogenesis of neurodegenerative disorders, especially AD. An increasing number of studies report that gut microbiomes are involved in multiple features of AD, such as cognitive impairment, hippocampal Aβ plaques, destroyed neuronal integrity, and plasticity and inflammation (Fang et al., 2020; Ma J. et al., 2020; Wang et al., 2020). Although the lack of effective targeted therapeutic treatment in the pathogenesis of AD, EA was considered a promising way to attenuate AD-related symptoms. The current widely accepted understanding is that EA reduces cognition impairment *via* anti-neuroinflammation Cai et al. (2019). However, little is known regarding the effect of EA on the gut microbiota. Therefore, we performed the 16S rRNA gene sequencing to explore the effect of EA on cognitive impairment in AD model mice, PS cDKO mice, underlying the regulation of microbiota. The acupoints, HT7 and KI3, were required for memory improvement, coming from the Chinese Traditional Medicine of “ShenMing” therapy with amounts of clinical practices.

Amino acids are important in neurotransmission and receptor function and are related to neurotoxicity, and changes in amino acid metabolism can be an early indicator of neurodegeneration in AD (Fonteh et al., 2007). Neurotransmitters are the important parts of the nervous system, closely related to the learning and memory ability of organisms. Recent studies have indicated alterations in amino acid metabolism in AD (Kaddurah-Daouk et al., 2011; González-Domínguez et al., 2014). Glycine is one of the components for the synthesis of reduced glutathione whose deficiency promotes the pathogenesis of AD (Wu et al., 2004; Peter and Fau-Braidy, 2015).

As an agonist of the N-methyl-d-aspartate glutamate receptor (NMDAR), glutamate is a major excitatory neurotransmitter in the mammalian central nervous system (CNS) (Niciu et al., 2012). It also comes from glutathione mentalism. In the brain, it is used for energy formation and biosynthesis of the inhibitory mediator, γ-aminobutyric acid (GABA) (Patel et al., 2005). Glutamate and its receptors, primarily the ligand-gated ionotropic glutamate receptors (iGluRs), play fundamental roles in synaptic plasticity as well as in the underlying molecular mechanisms of learning and memory (Chang et al., 2020). They mediate most of the excitatory neurotransmissions in the mammalian CNS. Studies have shown that reduced plasma glutamate level in patients with AD is associated with cognitive impairment (Lin et al., 2017). Furthermore, another study found that decreased hippocampal glutamate in patients with mild cognitive impairment and AD was associated with episodic memory performance (Wong et al., 2020). L-threonate (threonic acid) is a naturally occurring sugar acid that is excreted in urine by approximately 10% (Sun et al., 2016). It is widely reported that threonic acid has effects on the CNS. For example, oral administration of L-threonate magnesium salt (L-TAMS) can upregulate NMDAR signaling, prevent the synaptic loss, reverse memory deficits in aged rats, and improve synaptic density and memory in APPswe/PS1dE9 mice (Slutsky et al., 2010; Li W. et al., 2014). Moreover, older adults aged 50–70 years with cognitive impairment who orally took a compound containing L-TAMS for 12 weeks showed restored cognitive function (Liu et al., 2016). Importantly, intake of other Mg (2+) anions did not have the same results. In our study, PS cDKO mice demonstrated significantly decreased threonic acid when compared with the WT controls, but EA significantly reversed the level of threonic acid. Therefore, EA may alleviate the symptoms of AD mice by increasing the threonic acid content. Though our study showed that EA reduced the increased concentration of isovalerylglycine in the urine of PS cDKO mice, the connection between isovalerylglycine and AD is not clear. Interestingly, it was reported lower kidney clearance of isovalerylglycine was associated with a long-term decline in cognitive function in people with chronic kidney disease, but its serum concentration did not show that it was related to the decline of cognitive ability (Cunnane et al., 2011).

Citric acid, cis-aconitate, and succinic acid are important components of the TCA cycle, which is involved in glucose metabolism. Glucose metabolism plays an important role in patients with AD. It has been reported that neuronal glucose metabolism decreases by 20–25% in patients with AD, limiting cellular metabolic capacity and leading to oxidative stress (Cunnane et al., 2011; Butterfield and Halliwell, 2019). The levels of those three metabolites in AD mice are much lower than that of WT mice, suggesting that the glucose metabolism was reduced in AD mice. Unfortunately, EA did not improve glucose metabolism in cDKO mice. Fructose is also an important energy substance and plays a completely different role in the occurrence and development of AD. There is evidence that threefold to fivefold higher cerebral sorbitol and fructose levels in patients with AD may be related to the production of endogenous fructose (Xu et al., 2016). Excessive activation of brain fructose metabolism will cause mitochondrial oxidative stress and local inflammation, and mitochondrial energy production will be hindered by insufficient glycolysis of neurons, resulting in the gradual loss of brain energy level required by neurons to maintain function and survival (Johnson et al., 2020). Butyrate is a kind of multifunctional molecule that regulates host energy metabolism and immune function by utilization as an energy source using the β-oxidation pathway and as an inhibitor of histone deacetylases (Ferrante et al., 2003; Kim et al., 2009; Govindarajan et al., 2011; Stilling et al., 2016). In addition, butyrate was also reported to reduce gut inflammation by decreasing the activities of the NF-κB and signal transducer and activator of transcription 3 (STAT3) pathways and promoting T-regulatory cell differentiation (Chen et al., 2019). In Figure 3B, butyrate levels were significantly increased in the cDKO + EA group compared with the cDKO group, therefore, we reasoned that the improvement of AD-related symptoms by EA might be related to the increase in butyrate. Similarly, a modified Mediterranean-ketogenic diet benefits AD and increases fecal propionate and butyrate in subjects with mild cognitive impairment, and butyrate correlated negatively with Aβ-42 in cerebrospinal fluid (Nagpal et al., 2019). Moreover, *in vitro* research found that pretreatment with butyrate in the amyloid beta (Aβ)-induced BV2 cells showed suppressed microglial activation, reduced expression of cyclooxygenase-2 (COX-2), and reversed phosphorylation of NF-κB p65 (Sun J. et al., 2020). The finding is consistent with our study, though the purpose focused on is different.

Our previous study demonstrated that PS cDKO mice showed altered microbiota and metabolites (Gao et al., 2021b). Furthermore, in this study, behavioral tests (Y maze and Novel object recognition task) demonstrated ameliorated cognitive deficits in PS cDKO mice after EA treatment (Figure 1). In addition, EA could modulate the imbalance of the gut microbiota in PS cDKO mice, showing increased richness and evenness in the microbiotic community (Figure 5).

Consistently with our previous study, PS cDKO mice displayed reduced enrichment of *norank_f_Muribaculaceae*, *Lachnospiraceae_NK4A136_group*, and *Mucispirillum* in the research. Importantly, *norank_f_Muribaculaceae* is positively associated with the formation and barrier function of the inner mucus layer in the gut (Volk et al., 2019) and is predicted to produce propionate in feces as a fermentation end product. Furthermore, *norank_f_Muribaculaceae* and propionate both have been linked with gut health and growing longevity in mice in previous studies (Sibai et al., 2020; Smith et al., 2021). Other studies about sepsis-related liver injury (SLI) demonstrated that Metformin improves liver damage, regulates colon barrier dysfunction, and reduces inflammation in aged SLI rats, accompanied by an increased proportion of *Muribaculaceae* (Liang et al., 2022). *Lachnospiraceae_NK4A136_group*, also a type of short-chain fatty acid (SCFA) producing bacterium, was considered to be correlated with enhanced gut barrier function (Ma L. et al., 2020). It was also observed to decrease in diet-induced obese mice and subsequently increased by spermidine (Ma L. et al., 2020). *Mucispirillum* affiliated with the phylum *Deferribacteres* is an immune-inducing bacterial group (Herp et al., 2021), but it also promotes health in the immunocompetent host. Research (Zhang et al., 2021) found that C57BL/6 mice with lipopolysaccharide (LPS)-induced intestinal injury showed an increased abundance of *Mucispirillum.* However, with the inflammatory responses in the jejunum and the colon, such as enhanced mRNA levels of Toll-like receptor 4 (TLR4), pro-inflammatory cytokines, and chemokines, attenuated after glycine administration, the relative abundance of *Mucispirillum* increased. Moreover, *Mucispirillum schaedleri*, a species affiliated with the genus *Mucispirillum*, was reported to protect mice against *Salmonella typhimurium*-induced colitis partially by competitive binding anaerobic electron acceptors and was important to intestinal homeostasis (Herp et al., 2019). In addition, transgenic mice that overexpress the tryptophan metabolizing enzyme indoleamine 2, 3-dioxygenase 1 (IDO1) showed twofold thicker intestinal mucus layers than control mice, with increased proportions of *Mucispirillum schaedleri* (Alvarado et al., 2019). Although it is hard to explain the reduced abundance of *Mucispirillum* in PS cDKO mice, EA significantly improved the abundance of it, which may interfere with the progress of AD (Figures 6E, F). On the other hand, besides PS cDKO mice, patients with functional constipation were also reported to have a significantly lower abundance of *Mucispirillum* (Sugitani et al., 2021). *Lactobacillus* species are attractive hosts because of their GRAS (generally recognized as safe) status (Peiroten and Landete, 2020). *Lactobacillus* and its derivatives possess effects of anti-biofilm, antioxidant, pathogen-inhibition, and immunomodulation activities (Slattery et al., 2019; Chee et al., 2020). A reasonable explanation for the increased *Lactobacillus* in PS cDKO mice could be that it is a response to immunomodulation activities, and a tauopathy mouse model, P301L mice, showed increased *Lactobacillus* in fecal samples as well (Sun et al., 2019).

Differential urinary metabolites were integrated with the gut microbiota at the genus level to point out the relationship between gut microbial and host metabolism, especially in PS cDKO mice treated by EA (Figure 7). As shown in Figure 4, glycine, serine, and threonine metabolism, and glyoxylate and dicarboxylate metabolism were the two main metabolic pathways disturbed by EA. The levels of glycine and threonic acid, which significantly increased after EA treatment, were both positively correlated with *Lachnospiraceae_NK4A136_group* and *Mucispirillum* (Figure 7), and the abundance of *Mucispirillum* was significantly improved as well, though its immunomodulation activities were still thought complex. As mentioned earlier, *Lachnospiraceae_NK4A136_group* is correlated with enhanced gut barrier function, and intake of L-TAMS upregulates NMDAR signaling, improves synaptic density, and reverses cognitive deficits. Therefore, we may speculate that *Mucispirillum* increased by EA is beneficial for the recovery of AD, and butyrate, as a key mediator of host–microbe crosstalk, restored intestinal mucosa damage induced by a high-fat diet, increased the expression of zonula occluden-1 in the small intestine, and further decreased the levels of gut endotoxin in serum and liver (Zhou et al., 2017; Tian et al., 2019). In addition, *Lactobacillus* can produce butyric acid in what seems like a virtuous circle. Anyway, the association with neurological diseases and related biomarkers needs to be further explored.

## 5. Conclusion

**FIGURE 8:**
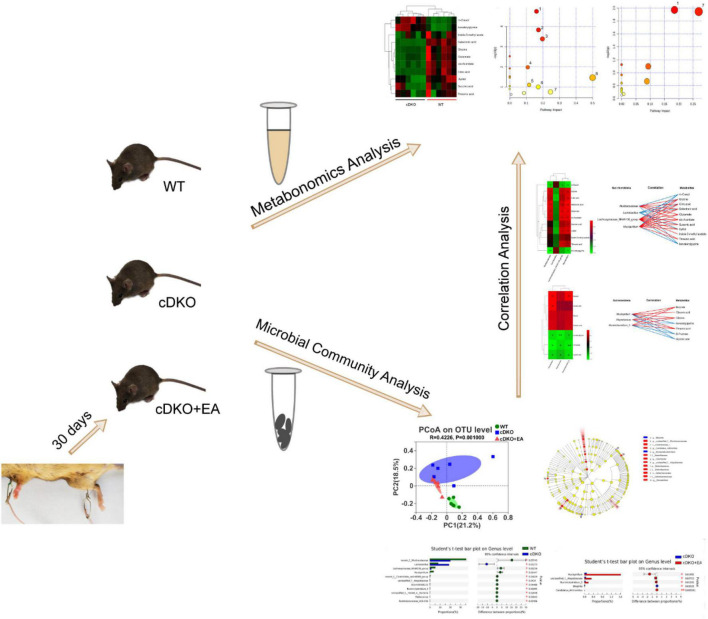
Schematic diagram representing the effect of electroacupuncture on urinary metabolome and microbiota in PS cDKO mice and correlation of gut microbiota and urinary metabolome.

Based on metabolomics and microbial community analysis, our study showed that EA alleviated cognitive deficits in PS cDKO mice along with altered urinary metabolites and gut microbiota (Figure 8). The EA treatment mainly disturbed two metabolic pathways: Glyoxylate and dicarboxylate metabolism glycine, serine, and threonine metabolism. Not only the decreased community diversity and richness but also the abundance of the main gut microbiome in PS cDKO mice was influenced by EA. Furthermore, differential urinary metabolites and gut microbiota were correlated. Differential urinary metabolites including increased isovalerylglycine and decreased glycine and threonine in PS cDKO mice were reversed by EA, as well as the differential gut microbiota, *Mucispirillum*. Based on previous studies, glycine benefits attenuating the inflammatory response and is an element for the synthesis of glutathione, the deficiency of which promotes the pathogenesis of AD. At the same time, threonine was proven to interfere with pathological manifestations of AD, such as improving synaptic density, upregulating NMDAR expression, and restoring cognitive function (Table 1). In our study, *Mucispirillum* was positively correlated with glycine and threonic acid (Figure 7) and could reduce intestinal inflammation (Table 1). The bidirectional crosstalk in the MGB axis contains the pathway of the immune system, and neuroinflammation in AD may be relieved with the modulation of systemic immunity. These may be the potential mechanism of EA improving cognitive ability in PS cDKO mice. Unfortunately, we did not measure the expression of these markers involved in the pathogenesis of AD, but it is a good way to study in future.

**TABLE 1:** Reversed metabolites and microbiome by electroacupuncture on PS cDKO mice and their collection with pathological manifestations of AD.

Reversed metabolites and microbiome by EA on PS cDKO mice		The collection between reversed metabolites/Microbiome and pathological manifestations of AD	Patient/Model	References
Increased metabolite	Glycine	is one of the components for the synthesis of glutathione whose deficiency promotes the pathogenesis of AD		Peter and Fau-Braidy (2015)
		attenuates the inflammatory responses in the jejunum and colon, such as enhanced mRNA levels of TLR4, pro-inflammatory cytokines	C57BL/6 mice with LPS-induced intestinal injury	Zhang et al. (2021)
	Threonic acid	increases synaptic density and upregulates NR2B-containing NMDAR expression	Sprague–Dawley rats; hippocampal neurons from rats; human neural stem cell-derived neurons	Sun et al. (2016)
		improves synaptic density and memory	APPswe/PS1dE9 mice	Li W. et al. (2014)
		restores cognitive function	older adults aged 50–70 with cognitive impairment	Liu et al. (2016)
Increased microbiome	*Mucispirillum*	is increased after Glycine administration with attenuated inflammatory responses	C57BL/6 mice with LPS-induced intestinal injury	Zhang et al. (2021)
		reduced *Salmonella* Typhimurium -induced gut inflammation partially by competitive binding anaerobic electron acceptors and is important to intestinal homeostasis	*Mucispirillum schaedleri*-colonized Oligo-MM^12^ mice	Herp et al. (2019)

## Data availability statement

The datasets presented in this study can be found in online repositories. The names of the repositories and accession numbers can be found at: https://www.ncbi.nlm.nih.gov/sra/PRJNA923176, PRJNA923176.

## Ethics statement

The animal study was reviewed and approved by the Animal Experimentation of Shanghai University of Traditional Chinese Medicine (permit number: PZSHUTCM191025005).

## Author contributions

JW, MZ, and YX accomplished the conception, design of the research, and approved final version of the manuscript. JG, NZ, ML, QW, and CZ performed the experiments. JG and NZ prepared the figures and analyzed and interpreted the data. JG, NZ, and ML drafted the manuscript. MZ edited and revised the manuscript. All authors read and approved the final manuscript.
