# Supplementary material for: Effects of electroacupuncture on urinary metabolome and microbiota in presenilin1/2 conditional double knockout mice
Source: Front Microbiol. 2023 Jan 24;13:1047121. doi: 10.3389/fmicb.2022.1047121 (PMC9904445; doi:10.3389/fmicb.2022.1047121)
Supplement: Supplementary file 1 [file Data_Sheet_1.docx]

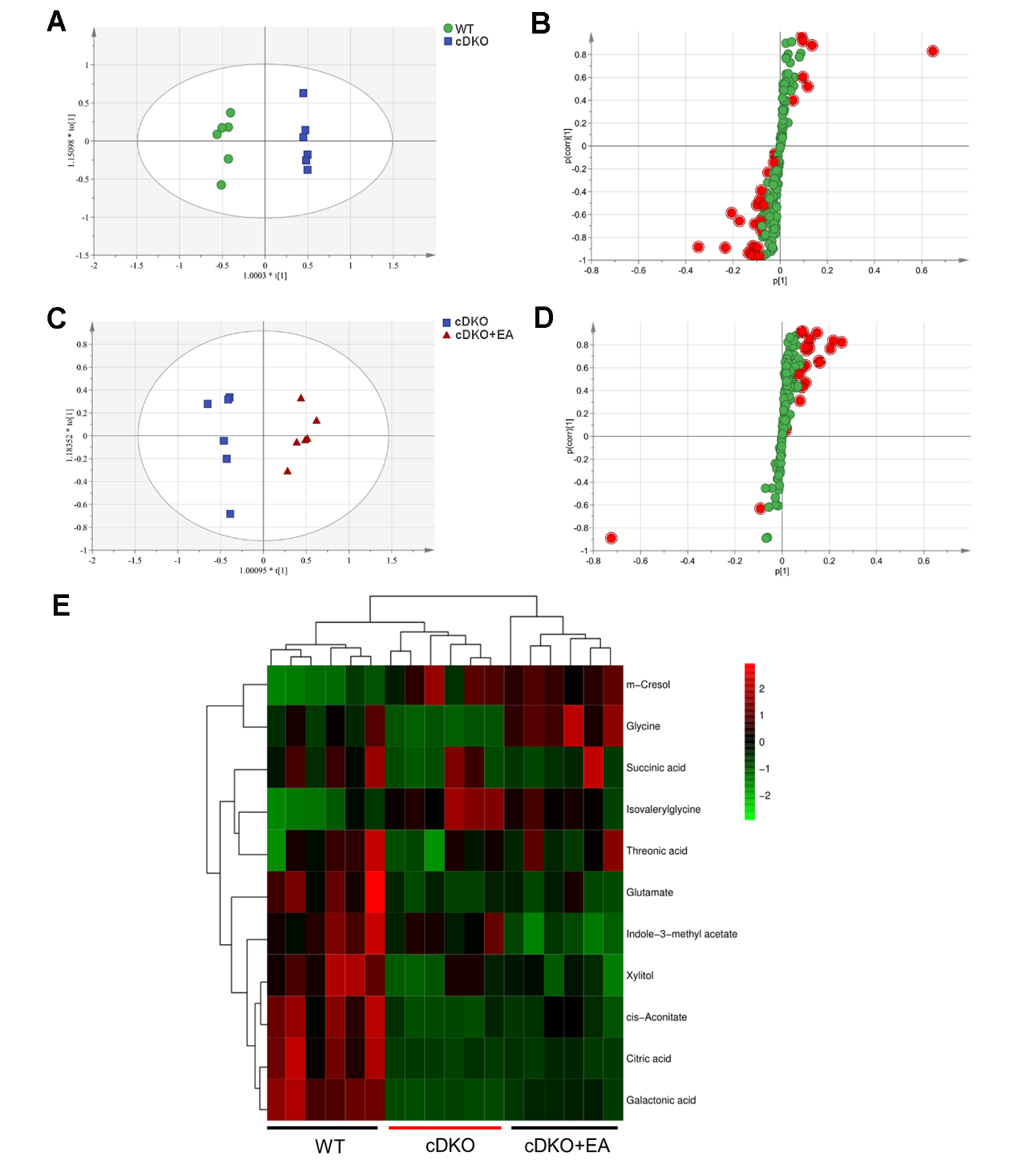


**Supplementary figure 1. Multivariate statistical analysis on urinary metabolites.** OPLS-DA score plots (A and C) and S-plots (B and D) between pairwise groups. (E) Heat map of the differential metabolites in WT, cDKO and cDKO+EA groups.

**Table S1 Differential metabolites between cDKO group and EA group.**

| Metabolites | RT (min) | Mass | cDKO *vs.* EA | | | Metabolic Pathways | Trend |
| --- | --- | --- | --- | --- | --- | --- | --- |
|  |  |  | Fold Change^a^ | VIP^b^ | t-test (*P*) |  |  |
| Butyrate | 5.66 | 117.0 | 8.83 | 4.42 | ＜0.05 | Others | ↓ |
| D-Fructose | 19.39 | 290.1 | 0.34 | 1.51 | ＜0.01 | Others | ↑ |
| Glycolic acid | 21.59 | 159.04 | 0.40 | 1.09 | ＜0.05 | Others | ↑ |
| Glyceric acid | 11.47 | 292.1 | 1.60 | 3.03 | ＜0.05 | Glyoxylate and dicarboxylate metabolism; Glycine, serine and threonine metabolism | ↓* |
| Glycine | 14.27 | 158.08 | 1.95 | 1.09 | ＜0.05 | Glyoxylate and dicarboxylate metabolism; Glycine, serine and threonine metabolism | ↓* |
| Isovalerylglycine | 15.32 | 172.1 | 0.72 | 1.77 | ＜0.001 | Others | ↑ |
| Threonic acid | 16.94 | 292.0 | 1.85 | 3.34 | ＜0.001 | Others | ↓ |

a Fold change was calculated as the ratio of the average relative level between the two groups (FC value = EA/cDKO).

b VIP was obtained from OPLS-DA with a threshold of 1.0.

* Metabolites involved in pathway analysis (impact factor ≥ 0.1)

↑ Metabolites increased in cDKO group

↓ Metabolites decreased in cDKO group

**Table S2 Differential metabolites between Control group and cDKO group.**

| Metabolites | RT (min) | Mass | WT *vs* cDKO | | | Metabolic Pathways | Trend |
| --- | --- | --- | --- | --- | --- | --- | --- |
|  |  |  | Fold Change^a^ | VIP^b^ | T-test (*P*) |  |  |
| cis-Aconitate | 21.26 | 147.0 | 2.28 | 1.88 | ＜0.001 | Citrate cycle (TCA cycle); glyoxylate and dicarboxylate metabolism | ↓* |
| Citric acid | 22.70 | 273.1 | 17.07 | 5.28 | ＜0.001 | Citrate cycle (TCA cycle); alanine, aspartate and glutamate metabolism | ↓* |
| m-Cresol | 7.51 | 165.0 | 0.18 | 1.05 | ＜0.05 | Others | ↑ |
| Galactonic acid | 26.28 | 293.1 | 7.55 | 1.21 | ＜0.001 | Others | ↓ |
| Glutamate | 17.36 | 198.0 | 6.02 | 1.06 | ＜0.001 | Glyoxylate and dicarboxylate metabolism; arginine biosynthesis; D-glutamine and D-glutamate metabolism; glutathione metabolism;alanine, aspartate and glutamate metabolism | ↓* |
| Glycine | 11.12 | 247.0 | 6.43 | 1.24 | ＜0.05 | glyoxylateanddicarboxylate metabolism; glutathione metabolism | ↓* |
| Indole-3-methyl acetate | 20.22 | 277.1 | 1.23 | 1.51 | ＜0.05 | Others | ↓ |
| Isovalerylglycine | 15.32 | 172.1 | 0.40 | 1.01 | ＜0.001 | Others | ↑ |
| Succinic acid | 11.13 | 147.0 | 1.67 | 1.75 | ＜0.05 | Citrate cycle (TCA cycle); alanine, aspartate and glutamate metabolism | ↓* |
| Threonic acid | 16.94 | 292.1 | 1.38 | 1.33 | ＜0.05 | Glyoxylate and dicarboxylate metabolism; Glycine, serine and threonine metabolism | ↓* |
| Xylitol | 20.51 | 217.1 | 1.54 | 1.23 | ＜0.01 | Others | ↓ |

a Fold change was calculated as the ratio of the average relative level between the two groups (FC value = WT/cDKO).

b VIP was obtained from OPLS-DA with a threshold of 1.0.

* Metabolites involved in pathway analysis (impact factor ≥ 0.1)

↑Metabolites increased in cDKO group

↓ Metabolites decreased in cDKO group
