# Supplementary material for: Effects of electroacupuncture on urinary metabolome and microbiota in presenilin1/2 conditional double knockout mice
Source: Front Microbiol. 2023 Jan 24;13:1047121. doi: 10.3389/fmicb.2022.1047121 (PMC9904445; doi:10.3389/fmicb.2022.1047121)
Supplement: Supplementary file 2 [file Data_Sheet_2.pdf]

Table S3 The duration and frequency of entries in novel arm of Y maze

no	duration (%)	frequency
WT-1	29.3392	31
WT-2	37.5219	32
WT-3	37.1969	40
WT-4	39.1634	27
WT-5	41.6215	33
WT-6	35.7054	31
cDKO-1	20.04	18
cDKO-2	25.4645	8
cDKO-3	29.9475	15
cDKO-4	26.3228	24
cDKO-5	31.4224	23
cDKO-6	29.3392	26
cDKO+EA-1	33.7972	32
cDKO+EA-2	29.5975	35
cDKO+EA-3	28.6059	34
cDKO+EA-4	30.6058	30
cDKO+EA-5	34.2055	27
cDKO+EA-6	35.2554	22

Table S4 Preference index in the novel object recognition task

no	training	1h test	24h test
WT-1	45.58824	62.58503	67.80105
WT-2	33.00781	60.37152	34.27896
WT-3	66.04478	76.1194	32.93413
WT-4	35.84184	59.80066	51.47059
WT-5	51.66667	65.49587	77.24719
WT-6	47.35226	66.88889	56.875
cDKO-1	41.93939	46.87898	50.64103
cDKO-2	64.31611	51.39665	46.92982
cDKO-3	36.09023	44.32749	33.98821
cDKO-4	37.26708	34.90099	51.91638
cDKO-5	37.26708	34.90099	51.91638
cDKO-6	63.11476	52.02703	71.60494
cDKO+EA-1	51.35323	54.12131	23.64217
cDKO+EA-2	47.87879	39.60396	40.42553
cDKO+EA-3	53.55649	64.99728	59.14333
cDKO+EA-4	42.13037	58.63248	74.54545
cDKO+EA-5	47.31405	46.67853	52.49501
cDKO+EA-6	39.82948	70.02342	38.7931

Table S5 Abundance of differential metabolites in WT and cDKO groups

Compound	Mass	RT	VIP	P	FC	WT-1	WT-2	WT-3	WT-4	WT-5	WT-6	cDKO-1	cDKO-2	cDKO-3	cDKO-4	cDKO-5	cDKO-6
m-Cresol	165.01	7.51	1.05	0.04	0.18	0.000138	0.000292	0.000753	0.00087	0.002267	0.0015	0.003088824	0.005208322	0.008407728	0.002591476	0.00652683	0.006274479
Glycine	247.03	11.12	1.24	0.02	6.43	0.002352	0.004865	0.001684	0.004354	0.002574	0.007211	0.00076875	0.000393245	0.000666003	0.00036214	0.000625344	0.00076829
Succinic acid	147.00	11.13	1.75	0.02	1.67	0.010802	0.021538	0.007976	0.019388	0.012021	0.031534	0.003883774	0.002128879	0.003343151	0.028165999	0.020180841	0.004051489
Isovalerylglycine	172.10	15.32	1.00	0.00	0.40	0.001094	0.001348	0.001456	0.001888	0.003283	0.002544	0.003706367	0.004051394	0.003484167	0.006320454	0.005824923	0.005742061
Threonic acid	292.10	16.94	1.33	0.03	1.38	0.005632	0.014862	0.013027	0.016824	0.016294	0.024837	0.009083963	0.009528247	0.005367929	0.015164359	0.012185667	0.014787714
Glutamate	198.00	17.36	1.06	0.00	6.02	0.002547	0.003564	0.001263	0.003173	0.001815	0.005929	0.00070816	0.000131663	0.0008029	0.000281029	0.000277307	0.00083779
Indole-3-methyl acetate	277.10	20.22	1.51	0.03	1.23	0.032034	0.028509	0.034715	0.044968	0.038741	0.054148	0.025807892	0.033765038	0.032726897	0.026362276	0.02967417	0.041592715
Xylitol	217.10	20.51	1.23	0.00	1.54	0.009747	0.011646	0.009889	0.014765	0.014716	0.012311	0.006395257	0.006230171	0.006473567	0.010138867	0.01012982	0.008233851
cis-Aconitate	147.00	21.26	1.88	0.00	2.28	0.01287	0.014706	0.008103	0.014008	0.010188	0.016252	0.006347812	0.004905419	0.005111725	0.005330657	0.00497066	0.006741076
Citric acid	273.10	22.71	5.28	0.00	17.07	0.067445	0.100563	0.031892	0.070897	0.039654	0.092755	0.004393786	0.003219922	0.00343374	0.001373724	0.006774688	0.004419234
Galactonic acid	293.10	26.28	1.21	0.00	7.55	0.005068	0.005793	0.003645	0.003818	0.004333	0.004378	0.00052581	0.000425721	0.000658335	0.000785736	0.000582305	0.00060162

Table S6 Abundance of differential metabolites in cDKO and cDKO+EA groups

Compound	Mass	RT	VIP	P	FC	cDKO-1	cDKO-2	cDKO-3	cDKO-4	cDKO-5	cDKO-6	cDKO+EA-1	cDKO+EA-2	cDKO+EA-3	cDKO+EA-4	cDKO+EA-5	cDKO+EA-6
Butyrate	117.00	5.66	4.42	0.02	8.83	0.003213	0.004081	0.003541	0.007211	0.001559	0.0075	0.024565592	0.037472878	0.024214248	0.035938131	0.043742557	0.073291411
Glyceric acid	292.10	11.47	3.03	0.03	1.60	0.000693	0.018451	0.040262	0.029459	0.020855	0.018565	0.029681192	0.035722738	0.038722735	0.027380259	0.037278301	0.036901671
Glycine	158.08	14.27	1.09	0.02	1.95	0.003495	0.005262	0.004419	0.001878	0.002572	0.005704	0.005840693	0.007256253	0.006441209	0.011330039	0.005084168	0.00956518
Isovalerylglycine	172.10	15.32	1.77	0.00	0.72	0.00429	0.007051	0.005396	0.00632	0.005825	0.005742	0.003863641	0.004735805	0.003598102	0.003891605	0.003613266	0.005352494
Threonic acid	292.10	16.94	3.34	0.00	1.85	0.009084	0.009528	0.005368	0.005422	0.007151	0.010682	0.01138923	0.018768785	0.01149949	0.010320041	0.014198558	0.021375215
D-Fructose	290.10	19.39	1.51	0.01	0.34	0.007733	0.011261	0.008257	0.017232	0.0115	0.014411	0.007247397	0.006440286	0.002263101	0.002993223	0.001727816	0.003578289
Glycolic acid	159.04	21.59	1.09	0.02	0.40	0.002655	0.003722	0.004378	0.004641	0.003963	0.00461	0.002331562	0.002342787	0.001326986	0.001532577	0.00104879	0.000948494

Table S7 The α-diversity indexes of gut microbiota

no	shannon	chao
WT-1	4.368057	564.27027
WT-2	4.254951	449.560976
WT-3	4.210651	483.75
WT-4	4.097182	543.666667
WT-5	3.879627	459.386364
WT-6	4.376759	515.468085
cDKO-1	2.521726	162.375
cDKO-2	3.443613	422.294118
cDKO-3	2.82105	330.166667
cDKO-4	4.107747	430.028571
cDKO-5	3.758691	448.076923
cDKO-6	3.962388	505.136364
cDKO+EA-1	4.038757	457
cDKO+EA-2	4.124781	511.022222
cDKO+EA-3	3.736269	461.5
cDKO+EA-4	3.998805	545.428571
cDKO+EA-5	3.681852	471.105263
cDKO+EA-6	4.37	503.394737

Table S8 The abundance of gut microbiome at and genus level

OTU ID	WT-1	WT-2	WT-3	WT-4	WT-5	WT-6	cDKO-1	cDKO-2	cDKO-3	cDKO-4	cDKO-5	cDKO-6	cDKO+EA-1	cDKO+EA-2	cDKO+EA-3	cDKO+EA-4	cDKO+EA-5	cDKO+EA-6
g__norank_f__Muribaculaceae	21652	17088	7303	11813	12620	12446	9011	10965	11052	6149	1144	3949	11625	8928	17757	9338	10409	9673
g__Lactobacillus	3700	3513	2799	3668	3474	1416	8845	5921	5434	9082	1153	9903	13552	16117	6069	10727	9826	6530
g__Bacteroides	493	2912	1883	3301	2165	1204	11256	352	909	1868	13976	11451	515	2012	376	1173	1245	2320
g__Dubosiella	1530	771	490	648	427	268	191	9740	2042	128	327	6	1147	891	1505	1176	6046	1307
g__Lachnospiraceae_NK4A136_group	553	1722	1904	2713	1112	4095	182	40	847	1139	0.1	428	954	403	543	818	1274	1774
g__Prevotellaceae_UCG-001	686	663	5834	513	1340	1701	1735	305	713	204	8	570	307	3282	31	313	111	440
g__Odoribacter	1259	886	1005	777	332	603	86	35	48	2360	27	250	3557	967	1419	2414	1054	1105
g__Parasutterella	3866	1005	1610	1174	139	857	461	748	2689	62	952	87	650	355	877	892	620	602
g__unclassified_f__Lachnospiraceae	200	456	1144	3182	2352	1542	968	47	467	886	41	197	235	273	456	1205	945	1149
g__norank_f__Desulfovibrionaceae	608	296	1309	1146	753	1939	440	19	1425	1426	961	248	458	182	691	641	469	255
g__Mucispirillum	171	1762	1447	1386	306	3067	13	15	61	63	24	14	298	357	1610	493	860	237
g__Parabacteroides	231	728	404	411	1627	343	915	155	236	45	2296	1060	224	680	47	1146	480	714
g__unclassified_f__Ruminococcaceae	2701	379	530	408	471	609	194	32	91	346	29	36	483	404	2475	486	462	123
g__Rikenella	1738	834	531	590	140	399	95	18	23	2604	0.1	419	344	340	384	787	346	405
g__Romboutsia	0.1	18	137	41	0.1	9	1325	1404	228	357	55	6	1739	554	319	430	748	577
g__Rikenellaceae_RC9_gut_group	610	117	148	161	129	161	659	58	110	682	0.1	39	768	484	58	547	1559	1538
g__Alistipes	496	203	88	250	52	215	79	5	91	2930	1	363	538	356	594	383	337	296
g__Helicobacter	315	107	2161	611	56	365	172	170	174	506	240	490	180	93	471	162	339	485
g__norank_f__Lachnospiraceae	172	276	494	651	291	513	397	10	404	890	0.1	673	206	277	335	272	551	521
g__Mycoplasma	1646	5	2604	45	182	218	119	35	2	53	9	390	11	22	667	39	69	0.1
g__Desulfovibrio	560	426	246	309	474	249	137	12	152	729	82	414	147	297	819	454	420	127
g__Erysipelatoclostridium	7	9	46	691	190	16	702	4224	5	17	5	4	0.1	5	0.1	13	1	2
g__Muribaculum	281	723	93	123	940	438	224	104	897	27	249	50	217	148	353	122	275	228
g__Lachnospiraceae_UCG-006	26	110	333	277	15	84	31	2	114	369	0.1	1339	277	536	299	293	361	404
g__Lachnoclostridium	48	62	211	567	299	256	346	24	226	426	847	198	204	252	142	145	219	276
g__Ruminiclostridium_9	174	166	220	329	285	152	197	52	163	384	0.1	708	172	327	387	317	350	246
g__Roseburia	55	243	616	1135	86	553	568	7	111	332	0.1	103	58	49	81	120	98	132
g__Enterorhabdus	202	176	215	163	68	82	137	84	26	364	20	297	228	234	309	551	161	193
g__Blautia	12	33	12	168	62	187	54	5	71	99	2276	29	39	36	29	79	49	37
g__Alloprevotella	18	12	97	70	188	623	362	347	784	9	45	0.1	112	88	19	8	34	159
g__Candidatus_Saccharimonas	34	368	255	113	19	66	4	3	37	529	0.1	416	93	343	78	348	55	118
g__unclassified_o__Bacteroidales	115	196	148	268	317	99	166	34	59	14	143	90	69	106	33	90	129	162
g__Anaeroplasma	55	289	1284	395	3	157	0.1	0.1	0.1	5	0.1	14	1	3	7	2	2	0.1
g__[Eubacterium]_xylanophilum_grou	0.1	319	16	32	1	67	25	0.1	90	169	0.1	9	147	53	256	90	225	106
g__Clostridioides	0.1	0.1	0.1	0.1	0.1	0.1	0.1	162	1	0.1	1231	0.1	0.1	0.1	0.1	0.1	0.1	0.1

g__Escherichia-Shigella	20	0.1	0.1	1	16	0.1	546	264	5	0.1	194	0.1	7	2	0.1	0.1	128	173
g__Anaerotruncus	106	6	33	71	323	93	223	30	14	41	3	47	22	24	83	38	92	82
g__unclassified_f__Atopobiaceae	200	54	52	48	38	60	37	37	14	37	9	1	162	75	287	120	80	16
g__A2	171	31	39	39	89	5	9	0.1	8	193	0.1	229	25	24	25	13	166	185
g__Ruminococcaceae_UCG-013	97	111	41	92	7	33	45	67	95	150	0.1	38	69	58	159	80	50	48
g__norank_f__Clostridiales_vadinBB60_group	77	270	208	177	13	98	0.1	0.1	5	46	0.1	66	39	31	54	60	25	16
g__norank_f__Peptococcaceae	7	15	86	307	27	220	23	1	54	65	0.1	7	3	3	9	51	61	155
g__[Clostridium]_innocuum_group	0.1	0.1	2	0.1	0.1	0.1	0.1	59	3	0.1	995	0.1	0.1	0.1	0.1	0.1	0.1	0.1
g__norank_f__Ruminococcaceae	12	71	61	141	36	97	131	5	42	51	0.1	6	3	16	17	72	28	85
g__Ruminiclostridium_5	95	39	87	87	7	61	38	2	6	34	0.1	15	28	33	52	117	83	81
g__Ruminococcaceae_UCG-014	27	342	28	50	15	48	7	2	58	69	0.1	42	7	38	13	86	5	8
g__norank_f__Erysipelotrichaceae	14	7	90	60	46	9	1	3	21	47	1	223	1	48	5	37	69	54
g__Oscillibacter	10	4	28	73	49	63	76	3	44	49	0.1	19	6	10	11	15	64	144
g__[Eubacterium]_brachy_group	30	19	23	25	21	12	18	6	27	117	0.1	74	52	68	35	71	23	24
g__unclassified_f__Muribaculaceae	26	73	11	19	94	62	35	33	156	1	24	1	24	9	27	3	18	15
g__GCA-900066225	133	5	12	216	88	39	35	2	1	7	1	0.1	5	5	4	38	2	4
g__Marvinbryantia	49	0.1	22	60	64	13	22	1	1	23	0.1	14	7	17	20	39	124	107
g__Ruminococcaceae_UCG-004	0.1	8	5	7	98	26	0.1	0.1	16	52	1	20	9	13	15	20	134	90
g__Streptococcus	43	6	16	35	35	7	17	27	6	51	3	54	15	31	13	49	57	47
g__Bifidobacterium	69	36	24	12	16	0.1	1	249	13	1	5	0.1	4	7	25	2	15	3
g__Negativibacillus	12	3	99	8	7	55	1	13	35	19	0.1	8	12	1	35	6	38	93
g__Lactococcus	34	1	41	22	11	3	19	0.1	1	34	0.1	10	8	26	15	108	33	57
g__GCA-900066575	7	3	39	34	3	81	11	3	11	13	0.1	15	0.1	5	1	31	22	74
g__Ruminiclostridium_6	0.1	24	2	15	5	6	8	0.1	0.1	113	0.1	2	36	24	33	27	27	18
g__Burkholderia-Caballeronia-Paraburkholderia	30	7	5	14	6	8	7	3	5	35	1	51	13	13	17	104	11	10
g__[Eubacterium]_nodatum_group	7	3	17	12	2	6	41	2	25	69	0.1	61	0.1	31	0.1	13	5	22
g__norank_f__norank_o__Mollicutes_RF39	2	79	26	38	5	8	0.1	0.1	7	29	0.1	15	4	14	0.1	43	11	14
g__norank_f__norank_o__Chloroplast	111	138	1	2	9	1	0.1	1	1	1	1	0.1	0.1	0.1	2	4	1	3
g__Bacillus	60	57	2	1	149	1	0.1	0.1	1	0.1	0.1	1	3	0.1	0.1	0.1	1	0.1
g__Coriobacteriaceae_UCG-002	7	18	7	3	4	6	6	169	16	2	5	0.1	3	0.1	6	4	3	4
g__Lachnospiraceae_UCG-001	45	4	12	3	2	4	0.1	0.1	25	16	0.1	1	8	4	9	3	58	61
g__Faecalibaculum	88	17	22	15	34	9	2	37	14	4	1	0.1	0.1	2	0.1	4	1	0.1
g__Intestinimonas	2	1	12	41	5	4	52	1	12	4	0.1	4	3	2	7	3	13	41
g__Family_XIII_UCG-001	5	0.1	1	8	5	4	1	0.1	15	43	0.1	16	8	5	6	2	15	15
g__unclassified_f__Prevotellaceae	0.1	3	13	6	33	6	24	2	9	0.1	0.1	0.1	8	18	0.1	21	1	1

g__Butyricicoccus	2	5	5	7	19	7	8	0.1	7	10	0.1	14	0.1	4	7	6	25	17
g__Rodentibacter	9	0.1	5	12	3	1	1	1	0.1	1	1	1	15	14	5	55	11	4
g__unclassified_f__Eggerthellaceae	9	3	5	7	11	6	8	1	1	19	0.1	6	11	7	15	12	7	10
g__Ruminiclostridium	1	2	6	14	3	21	11	0.1	4	14	0.1	11	1	1	5	4	10	26
g__Candidatus_Stoquefichus	2	3	81	9	1	0.1	2	2	0.1	9	0.1	0.1	0.1	2	0.1	9	1	7
g__ASF356	1	0.1	7	7	13	11	23	0.1	9	6	0.1	0.1	3	1	4	1	18	17
g__Weissella	0.1	0.1	23	21	0.1	66	0.1	0.1	0.1	0.1	0.1	0.1	0.1	1	0.1	1	0.1	0.1
g__Bilophila	12	6	7	7	6	12	13	0.1	3	18	11	2	0.1	0.1	0.1	0.1	5	0.1
g__Ruminococcaceae_NK4A214_group	5	16	15	12	2	9	0.1	0.1	2	21	0.1	2	3	3	1	2	3	4
g__Tyzzerella	0.1	0.1	5	15	2	6	4	0.1	15	2	0.1	0.1	3	0.1	3	1	9	21
g__Staphylococcus	0.1	2	11	2	3	8	15	1	0.1	20	0.1	0.1	4	3	2	6	3	6
g__Acetatifactor	0.1	1	5	29	0.1	4	0.1	0.1	9	3	0.1	2	1	0.1	0.1	1	5	19
g__[Ruminococcus]_torques_group	0.1	0.1	4	47	0.1	6	0.1	4	1	0.1	0.1	0.1	0.1	7	0.1	9	0.1	0.1
g__Christensenellaceae_R-7_group	0.1	4	8	19	0.1	3	0.1	0.1	8	0.1	0.1	3	0.1	1	0.1	20	0.1	0.1
g__Harryflintia	7	0.1	4	1	7	16	2	0.1	3	4	0.1	4	0.1	0.1	2	4	4	5
g__unclassified_p__Firmicutes	1	0.1	1	2	0.1	0.1	10	32	4	0.1	2	0.1	1	0.1	0.1	0.1	5	3
g__Lachnospiraceae_FCS020_group	0.1	0.1	1	10	0.1	0.1	1	0.1	2	3	0.1	4	1	0.1	0.1	2	9	27
g__Ralstonia	7	0.1	0.1	1	0.1	1	2	1	1	15	1	5	1	3	4	16	0.1	1
g__Ruminococcaceae_UCG-010	0.1	3	3	6	0.1	4	0.1	0.1	31	3	0.1	0.1	0.1	0.1	0.1	2	0.1	1
g__Ruminococcaceae_UCG-009	0.1	0.1	3	8	0.1	3	0.1	0.1	19	5	0.1	0.1	0.1	0.1	3	1	0.1	8
g__unclassified_k__norank_d__Bacteri	2	2	4	5	4	8	0.1	2	3	0.1	0.1	0.1	0.1	1	1	1	8	3
g__Corynebacterium	1	1	0.1	1	3	0.1	3	0.1	0.1	5	0.1	0.1	0.1	0.1	0.1	4	15	7
g__Enterococcus	0.1	0.1	5	0.1	0.1	0.1	4	6	0.1	0.1	20	0.1	2	1	1	0.1	0.1	1
g__Gordonibacter	6	5	2	7	0.1	2	3	3	1	0.1	2	0.1	0.1	3	2	2	1	0.1
g__Allobaculum	0.1	3	8	18	0.1	0.1	0.1	0.1	0.1	0.1	0.1	0.1	0.1	1	0.1	7	0.1	0.1
g__unclassified_f__Peptostreptococcac	0.1	0.1	0.1	0.1	0.1	1	2	6	4	0.1	0.1	0.1	8	3	2	1	3	7
g__Parvibacter	1	3	5	5	0.1	1	1	0.1	0.1	2	0.1	1	0.1	5	3	5	1	1
g__norank_f__Eggerthellaceae	2	1	1	1	2	0.1	2	0.1	0.1	0.1	1	2	3	0.1	6	9	4	0.1
g__norank_f__Neisseriaceae	1	0.1	1	1	1	1	1	0.1	0.1	8	0.1	6	1	4	1	1	4	0.1
g__Pseudomonas	25	0.1	0.1	2	0.1	0.1	0.1	0.1	0.1	0.1	0.1	0.1	0.1	0.1	0.1	0.1	0.1	0.1
